# Sensory Issues and Their Impact Among Autistic Children: A Cross-Sectional Study in Northern Sri Lanka

**DOI:** 10.7759/cureus.72130

**Published:** 2024-10-22

**Authors:** Sabarththiny Sivapalan, Bhavana Sivayokan, Kounthini Raveenthiran, Sambasivamoorthy Sivayokan

**Affiliations:** 1 Department of Psychiatry, University of Jaffna, Jaffna, LKA; 2 Department of Psychological Sciences, Kansas State University, Manhattan, USA; 3 Mathavam - Center for Neurodevelopmental Disorders, Regional Directorate of Health Services, Jaffna, LKA; 4 Mental Health Unit, Teaching Hospital Jaffna, Jaffna, LKA

**Keywords:** autism, autism spectrum disorder, childhood autism rating scale, hyperreactivity, hyporeactivity, sensory integration, sensory processing

## Abstract

Background: Sensory processing issues are among the key diagnostic criteria for autism spectrum disorder (ASD). As altered sensory processing causes autistic children to react differently to sensory experiences and has a profound impact on their development, affecting their learning ability, social interaction, and ability to adapt to a new environment, there is a need to recognize and address these issues in children diagnosed with ASD during assessments and interventions. This study aimed to identify the patterns of sensory issues and their impact, and selected correlates among autistic children attending a center for neurodevelopmental disorders in northern Sri Lanka.

Methods: This institution-based, descriptive, cross-sectional study was conducted at a center for neurodevelopmental disorders in Jaffna among 100 children diagnosed with ASD. The sociodemographic details of the child, and scores of the Childhood Autism Rating Scale second edition (CARS™ 2), Sensory Profile™ 2, and a locally developed Behavioral Checklist were extracted from the records available at the center. Data were analyzed using R statistical computing software (R Foundation for Statistical Computing, Vienna, Austria) using general linear models.

Results: All the children in this study had at least one sensory issue, with 50% having visual processing issues. The severity of ASD increased as auditory processing issues increased. Behavioral issues, in general, increased significantly with increasing auditory and visual processing issues. Repetitive behaviors significantly increased with increasing auditory processing issues, while problems with self-regulation increased significantly with increasing visual and movement processing issues. Conduct-related issues were found to increase significantly with increasing movement and visual processing issues, and attentional response issues were found to increase significantly with increasing auditory, visual, and touch processing issues.

Conclusion: The high prevalence of sensory issues in autistic children and its impact on the severity of ASD and behavioral issues are reiterated in this study. These results emphasize the importance of including interventions targeting sensory issues with the routine therapy for ASD.

## Introduction

The prevalence of autism spectrum disorder (ASD), which was 0.62% in 2012 [1] has increased to 1% within the span of a decade [2]. Autistic children exhibit poor social interactions, impaired communication, stereotyped behavior, and sensory issues, which are all included in the Diagnostic and Statistical Manual of Mental Disorders (DSM-5) diagnostic criteria for ASD [3]. While several studies around the world have shown the prevalence of sensory issues among children with autism to range from 90%[4] to 98%[5], a previous study conducted in Jaffna has shown this percentage to be only 78% [6]. 

Sensory issues among autistic children merit detailed study as they are known to have a significant positive association with the severity of ASD [7], as well as other symptoms of ASD, including communication skills, social relations, and repetitive behavior [8-10]. Altered sensory processing has a profound impact on the development of the child with autism, affecting their learning ability, social interaction, and ability to adapt to a new environment [11]. Therefore, there is a need to recognize and address these issues in autistic children during assessments and interventions.

Autistic individuals react differently to sensory experiences [12]. Sensory issues can be expressed as hyperreactivity (over-responsiveness) or hyporeactivity (under-responsiveness) and can be associated with a wide array of sensory stimuli, including sights, sounds, smells, tastes, touch, movements, and body positions. Based on an individual’s neurological threshold for sensory stimuli and their responses to such stimuli, sensory issues can be categorized into four quadrants, namely sensory sensitivity, sensory seeking, sensory avoiding, and low registration [13]. Individuals with sensory sensitivity and sensory avoidance both have a low threshold for sensory input. However, while those with sensory avoidance actively take steps to avoid sensory input (covering eyes/ears, running away from the source of stimuli), those with sensory sensitivity do not avoid the stimuli, instead expressing their distress through other behaviors. Similarly, individuals with low registration and sensory seeking have a high threshold for sensory stimuli, and while seekers actively pursue more sensory input, those with low registration do not.

As the sensory needs of each individual are unique, it is essential that they undergo sensory assessment using a standardized sensory tool, the results of which should be used to set individualized therapeutic goals. Furthermore, there is a need to understand the pattern of sensory issues among autistic children of a specific region and socio-cultural background, for the therapists to design effective interventions that better suit their client population. This study aimed to identify the patterns of sensory issues, their impact, and selected correlates among autistic children attending a center for neurodevelopmental disorders in northern Sri Lanka.

## Materials and methods

Study setting and participants 

This institution-based, descriptive, cross-sectional study was conducted at Mathavam - a center for neurodevelopmental disorders, which is under the purview of the Department of Health of the Northern Province, Jaffna, Sri Lanka, and functions as a key exemplar of public-private partnership models in health [14]. All procedures were approved by the Ethics Review Committee of the Faculty of Medicine, University of Jaffna (approval number: J/ERC/22/143/NDR/0285). Informed written consent was obtained from the parents before data collection.

Data were collected from 100 children diagnosed with ASD who attended the center for sensory assessment over a period of 18 months. Only the children who underwent sensory assessment for the first time were included in the study. Those who underwent sensory interventions elsewhere before sensory assessment at this center were excluded as their assessment would not reflect the pattern of sensory issues they originally presented with. Data were collected from all the children fulfilling the inclusion criteria.

Study instruments

The sociodemographic details of the child, and scores of the Childhood Autism Rating Scale second edition (CARS™ 2) [15], Sensory Profile™ 2 [16], and a locally developed Behavioral Checklist (see Appendices) were extracted from the records available at the center. 

CARS 2 is a widely used 15-item clinician rating scale that helps determine ASD symptom severity through quantifiable ratings based on direct observation of the child by a professional familiar with ASD who had also obtained training on rating CARS 2 items. 

Sensory Profile 2 is a standardized family of assessments that evaluates a child’s sensory processing patterns in the context of home, school, and community-based activities. This widely used tool is completed by caregivers, who are in the strongest position to observe the child’s responses to sensory interactions that occur throughout the day, and analyzes a child’s sensory processing issues related to various modalities, along with the behavioral responses associated with sensory processing. This assessment is different for children of different age groups. In the Sensory Profile 2 for children aged 7-35 months, general sensory processing, along with auditory, visual, touch, movement, and oral sensory processing are analyzed, while the Sensory Profile 2 for children aged 3-15 years assesses auditory, visual, touch, movement, body positioning, and oral sensory processing. This tool assesses only the general behavioral responses in children aged 7-35 months while assessing conduct, social-emotional responses, and attentional responses in children aged 3-15 years. These age-based versions of this tool not only have different components but also have different ranges of scores and cutoff values for classification. 

The Behavior Checklist is a parent-rated questionnaire used by the center to understand the behavioral issues of the child and contains six sections: social communication, restrictive behaviors, mood and anxiety, self regulation, challenging behavior, and self-injurious behavior. It was designed by the center incorporating key elements of widely-used behavior assessment tools tailored to the local context, and was content validated by local experts.

Data analysis 

Data was analyzed in R statistical computing software [17] using general linear models. Summary statistics were run to obtain the pattern of sensory issues present in the sample. Following that, multiple regressions were conducted to determine the effect of different sensory modalities on the severity of ASD and behavioral issues, as indicated by the scores of CARS 2 and Behavior Checklist, respectively. Since the number of children in the 7-35 months age group was not sufficient to examine all sensory modalities, the regression models were conducted only on the data of children aged 3-15 years.

## Results

General characteristics of the sample

The total number of children included in the study was 100. The mean age of the children was four years and five months (*SD*=18 months). The sample was predominantly of the male gender (79%). Only 8% of the children had a family history of ASD. Most of the children (62%) were from Jaffna, the district where the center is located, while 19% of children were from other districts of the country, and another 19% were children of the diaspora community. These characteristics are summarized in Table 1.

**Table 1:** General details of participants (N=100) Mean age was 4.5 years (*SD* = 18 months) NOTE: Frequency values are the same as the percentage as the sample size is 100

General characteristics	Percentage
Sex	
Male	79
Female	21
Family history of Autism Spectrum Disorder	
Yes	8
No	92
Geographic location	
Jaffna district	62
Other districts of Sri Lanka	19
Other countries	19

Sensory profile

Sensory Profile 2 consists of several sections that can be grouped into two main parts: sensory modalities and behavioral responses to sensory stimuli. Individual statements from these sections are assigned to one of four sensory quadrants and are summed to obtain the sensory quadrant score. Based on cutoff values determined by the normal curve and Sensory Profile 2 classification system [16], all sensory modalities, behavioral responses, and sensory quadrants are categorized into groups as being much less than, less than, just like, more than, or much more than the majority of children in the same age group. Table 2 shows this classification for this study sample, with classifications made according to the age-group-specific cutoff scores. When assessing the sensory issues of each child in the study, it was found that all 100 children had at least one sensory issue, defined as a score that falls into the ‘much less than’, ‘less than’, ‘more than’, or ‘much more than’ categories in the above classification system.

**Table 2:** Classification of sensory modalities, behavioral responses, and sensory quadrants NOTE: The Sensory Profile 2 categorizes all sensory modalities, behavioral responses, and sensory quadrants into groups as being much less than, less than, just like, more than, or much more than the majority of others in the same age group. The cutoff scores used in this classification system are different for each age groups. This table shows the classification according to the age group-specific cutoff scores. General processing and general behavioral responses are sections found only in the Sensory Profile 2 for 7-35-month-old children, whereas body position, conduct, social-emotional responses, and attentional responses are sections found in the Sensory Profile for 3-15-year-old children.

Sections of Sensory Profile 2	Much less			Less		Just like		More		Much more	
	Frequency	Percentage		Frequency	Percentage	Frequency	Percentage	Frequency	Percentage	Frequency	Percentage
Sensory modalities											
General processing (n=18)	-	-		-	-	5	27.78	-	-	13	72.22
Auditory (n=100)	-	-		3	3.00	79	79.00	6	6.00	12	12.00
Visual (n=100)	-	-		42	42.00	50	50.00	5	5.00	3	3.00
Touch (n=100)	-	-		-	-	70	70.00	24	24.00	6	6.00
Movement (n=100)	2	2.00		7	7.00	63	63.00	22	22.00	6	6.00
Body positioning (n=82)	-	-		-	-	78	95.12	2	2.44	2	2.44
Oral processing (n=100)	-	-		-	-	66	66.00	27	27.00	7	7.00
Behavioral responses											
Conduct (n=82)	-		-	-	-	49	59.76	30	36.59	3	3.65
Social-emotional responses (n=82)	-		-	-	-	49	59.76	25	30.49	8	9.75
Attentional responses (n=82)	-		-	-	-	46	56.10	27	32.92	9	10.98
General behavioral responses (n=18)	-		-	-	-	4	22.22	6	33.33	8	44.45
Sensory quadrants											
Seeking (n=100)	12		12.00	2	2.00	67	67.00	16	16.00	3	3.00
Avoiding (n=100)	-		-	-	-	76	76.00	18	18.00	6	6.00
Sensitivity (n=100)	-		-	-	-	49	49.00	42	42.00	9	9.00
Low registration (n=100)	-		-	-	-	85	85.00	12	12.00	3	3.00

CARS 2 and Behavior Checklist scores

The severity of ASD was measured using CARS 2, which has a maximum possible score of 40. The mean CARS 2 score of the children in this study was found to be 33.16 (*SD*=2.28). The mean score of the behavior checklist was 89.18 (*SD*=16.47).

Of the six sections of the Behavior Checklist, namely (i) social communication issues, (ii) repetitive behavior, (iii) mood- and anxiety-related behavior, (iv) self-regulation issues, (v) other challenging behaviors, and (vi) self-injurious behavior, the first has a total possible score of 60, while the remaining five sections each have a total possible score of 30, bringing the total possible score to 210. The mean scores of these sections were 30.72 (*SD*=7.10 ), 12.24 (*SD*=3.08), 12.42 (*SD*=4.61), 17.33 (*SD*=4.13), 9.92 (*SD*=3.74), and 6.55 (*SD*=1.00), respectively. 

Impact of sensory processing issues on the severity of ASD

**Figure 1:**
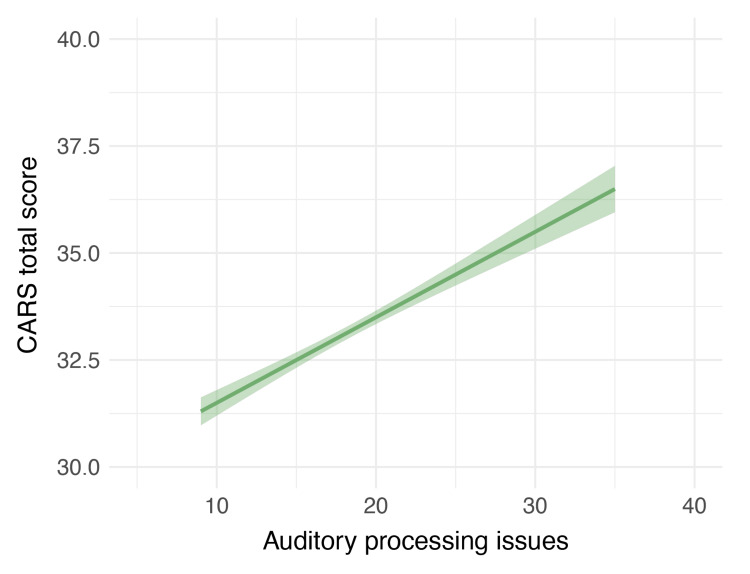
The effect of auditory processing issues on severity of ASD The values represented here are the fitted values from the statistical model, with the error ribbons representing standard error. As auditory processing issues increase, the Childhood Autism Rating Scale (CARS) scores also increase, indicating greater severity of ASD. ASD: autism spectrum disorder

Multiple regression was conducted to determine the effect of sensory processing issues on CARS 2 scores among children aged 3-15 years. The results showed that the model significantly accounted for a proportion of variance in ASD severity, *R^2^* =0.16, *F*(6,75)=3.65, *p*=.003. Examination of unique effects indicated that auditory processing issues, *b*=0.14, *SE*=0.06, *t*(75)=2.18, *p*=.033, was a significant predictor of ASD severity, such that as auditory processing issues increased, the severity of ASD also increased significantly (Figure 1).

Impact of sensory processing issues on behavioral responses

Sensory Profile 2 for children aged 3-15 years explores behavioral responses to sensory issues. Three multiple regression models were run to determine the effect of sensory processing issues on conduct, social-emotional response, and attentional response. The multiple regression model exploring the effect of sensory processing issues on conduct resulted in a significant model, *R^2^*=0.42, *F*(6,75)=10.61,* p*<.001, with examination of unique effects showing that movement processing issues, *b*=0.53, *SE*=0.09, *t*(75)=5.56, *p*<.001, and visual processing issues, *b*=0.30, *SE*=0.12, *t*(75)=2.43, *p*=.017, were significant predictors of conduct, such that as movement and visual processing issues increased, issues with conduct also significantly increased.

**Figure 2:**
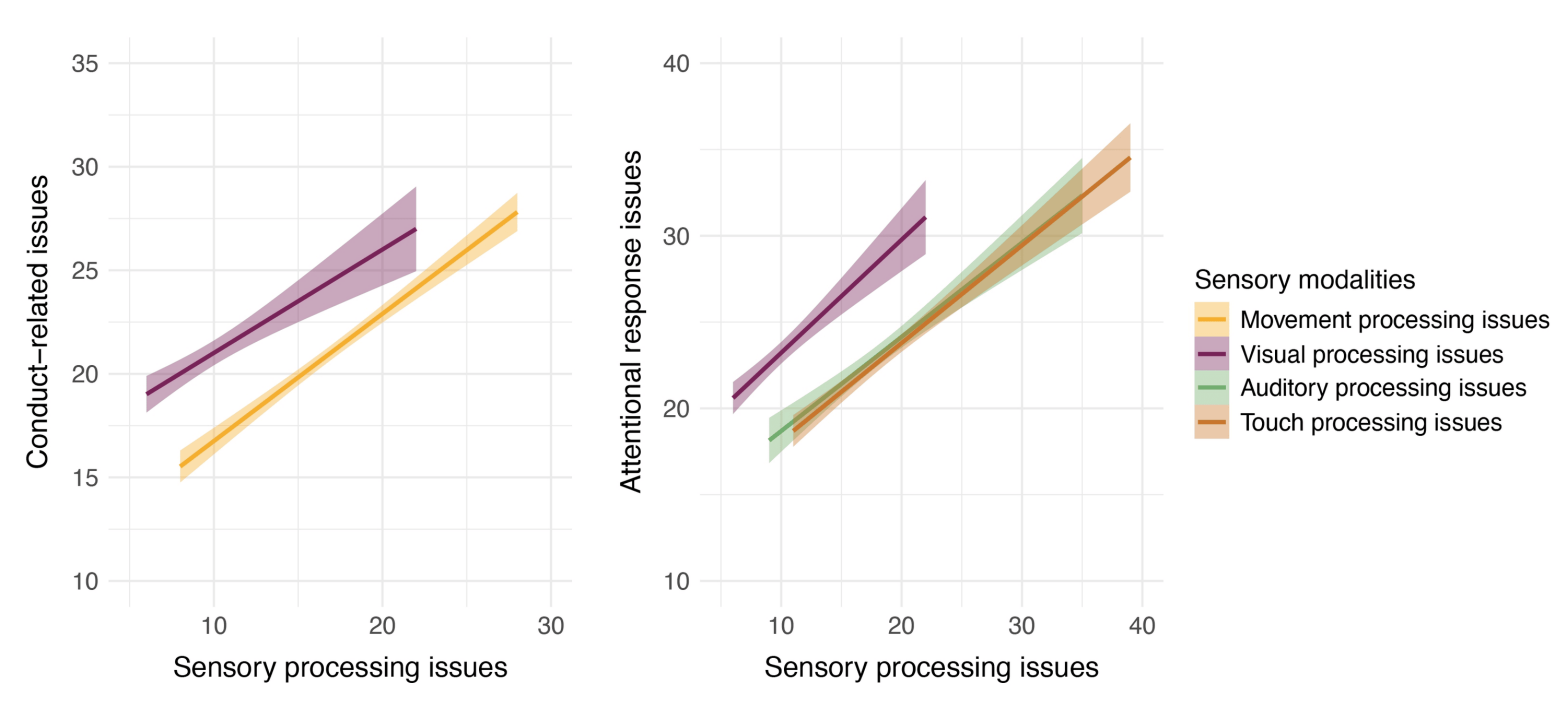
The effect of sensory processing issues on behavioral responses The values represented here are the fitted values from the statistical model, with the error ribbons representing standard error. The figure shows the effect of sensory processing issues on conduct (left) and attentional responses (right). Conduct-related issues appear to increase with increasing visual and movement processing issues, while attentional response issues appear to increase with increasing auditory, visual, and touch processing issues.

A multiple regression model determining the effect of sensory processing issues on social-emotional response did not yield a significant model. However, the model exploring the effect of sensory processing issues on attentional response was significant *R^2^*=0.32, *F*(6,75)=7.43, *p*<.001, with examination of unique effects indicating that auditory, *b*=0.36, *SE*=0.14, *t*(75)=2.53, *p*=.014, visual, *b*=0.37, *SE*=0.17,* t*(75)=2.17, *p*=.033, and touch, *b*=0.39, *SE*=0.13, *t*(75)=3.01, *p*=.004, processing issues were significant predictors of conduct, such that as processing issues in these sensory modalities increased, issues with attentional response also increased significantly (Figure 2). 

Impact of sensory processing issues on the Behavior Checklist

Multiple regression was conducted to determine the effect of sensory processing issues on the total score of the Behavior Checklist in children aged 3-15 years. This model accounted for a significant proportion of the variance of Behavior Checklist scores, *R*^2^=0.35, *F*(6,75)=8.39, *p*<.001. Examination of unique effects revealed that auditory, *b*=0.97, *SE*=0.38, *t*(75)=2.53, *p*=.014, and visual, *b*=1.21, *SE*=0.46, *t*(75)=2.65, *p*=.009, processing issues were significant predictors of behavioral issues, such that as auditory and visual processing issues increased, the total score for Behavior Checklist also increased significantly, indicating a significant overall increase in behavioral issues. 

**Figure 3:**
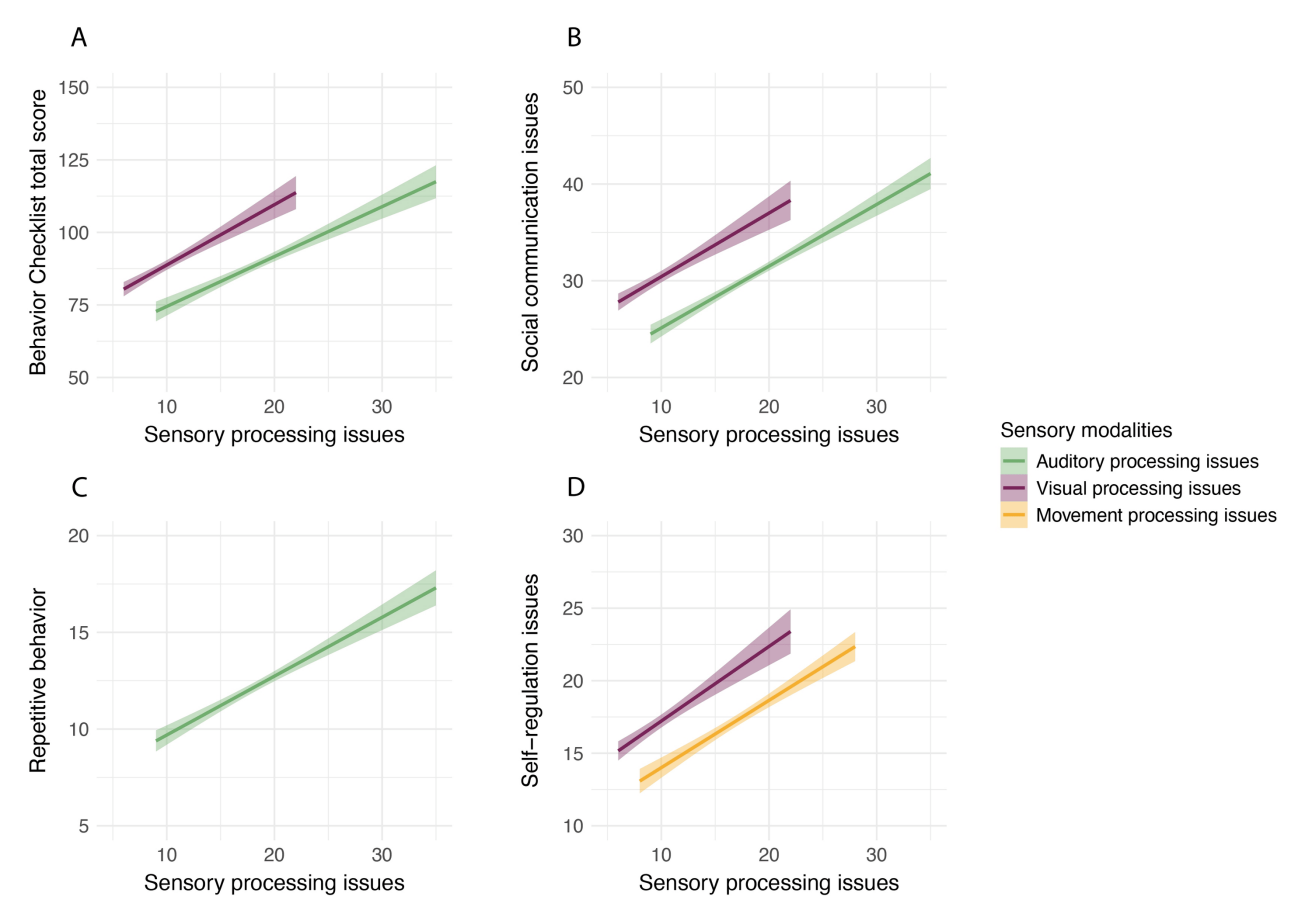
The effect of sensory processing issues on Behavior Checklist outcomes The values represented here are the fitted values from the statistical model, with the error ribbons representing standard error. The figure shows the effect of sensory processing issues on the total score of the Behavior Checklist (A), social communication issues (B), repetitive behavior (C), and self-regulation issues (D). The total score of the Behavior Checklist increases significantly with increasing auditory and visual processing issues. Social communication and repetitive behavior appear to be increasing with increasing auditory processing issues, while social communication issues and self-regulation issues are rising with increasing visual processing issues. Self-regulation issues also appear to increase significantly with increasing movement processing issues.

Multiple regression models were conducted to determine the effect of sensory processing issues on individual sections of the Behavior Checklist. Results showed that sensory processing issues accounted for a significant proportion of variance in social communication issues, R^2^=0.23, F(6,75)=4.94, p<.001, repetitive behavior, *R*^2^=0.28, *F*(6,75)=6.21, *p*<.001, and self-regulation issues, *R*^2^=0.39, *F*(6,75)=9.81, *p*<.001. Examination of unique effects in these models indicated that auditory, *b*=0.47, *SE*=0.17, *t*(75)=2.82, *p*=.006, and visual, *b*=0.43, *SE*=0.20, *t*(75)=2.12, *p*=.037, processing issues were significant predictors of social communication issues; auditory processing issues, *b*=0.17, *SE*=0.08, *t*(75)=2.22, *p*=.029, was a significant predictor of repetitive behaviors; visual, *b*=0.34, *SE*=0.11, *t*(75)=3.09,* p*=.003, and movement, *b*=0.35, *SE*=0.08, *t*(75)=4.19, *p*<.001, processing issues were significant predictors of self-regulation issues. In all these instances, as the sensory processing issues increased, the relevant behavioral issues increased significantly (Figure 3). 

## Discussion

The male-to-female ratio of autistic children in this study was approximately 4:1, which corresponds to the established ratio in the literature [18]. Early diagnosis of ASD is crucial as it not only enables the implementation of tailored interventions that meet the needs of each autistic individual but also improves long-term outcomes, including developmental outcomes and adaptive skills [19]. The average age of the children in this study, four years and five months, is reflective of the age of presentation to the center and indicates that these children sought help from an autism center much earlier than children in other South Asian countries [6]. However, it is not early enough to reap the maximum benefits of interventions [19]. 

A striking finding in this study is the presence of at least one sensory issue in all children. Significant sensory processing issues can make the world seem very different, sometimes to the extent of appearing altered or confusing [11]. This can cause children with sensory issues to sometimes become muddled and frightened, and experience breakdowns. Children with poor sensory regulation demonstrate a wide variety of difficulties across many domains including behavior problems, difficulties in emotional and attention regulation, and difficulties in daily activities. Sensory over-responsivity has been shown to be highly associated with early behavior problems and poorly developed adaptive social behaviors [20]. However, such signs of sensory overload may often be perceived as problematic behavior by parents. This misunderstanding coupled with failure to fulfill the sensory needs of the children may compound the problem, leading to more behavioral issues [20].

While sensory processing issues are not unique to ASD [21,22], they are a key feature of ASD. They are intertwined with the severity of ASD and its other features [7-10,23], and are suggested to be the root cause of problems such as language delay and difficulty recognizing emotions [24]. This is clearly reflected in this study, which shows greater sensory issues to be significant predictors of greater severity of ASD, and increased behavioral problems in different domains, such as conduct, attentional responses, social communication, repetitive behavior, and self-regulation-related behavior. However, it is important to note that these results do not specify any causal relationships. The results of this study align with what is revealed in the literature. Of all sensory modalities, auditory processing issues are perhaps the most studied, and are thought to be associated with language- and communication-related issues faced by autistic individuals [25]. This is reflected in the results of the present study, which shows auditory processing issues to be significantly associated with social communication issues. One of the commonly studied visual processing issues in ASD is that of face processing [26], which might explain why visual processing issues were found to be significant predictors of conduct, attentional responses, as well as social communication issues. Attention is involved in multiple stages of sensory processing. Therefore, abnormalities in the cortical circuitry mediating attention in autistic individuals could impact their abilities to attend to, process, and respond to sensory information [24,27]. This is reflected in the present study, where auditory, visual, and touch processing issues are shown to be significantly associated with attentional responses. 

The entwining nature of sensory issues also indicates that other interventions may not work efficiently if sensory issues are not identified and addressed in tandem. While the primary goal of sensory interventions is to improve sensory modulation, studies have shown other positive outcomes as well, such as enhanced self-regulation, better social interactions, increased attention, and enhanced participation in daily activities [28]. The literature identifies multiple interventions targeting sensory processing issues [23], with each intervention showing improvement in different sensory modalities. Finding the right intervention for the pattern of sensory issue a child has can be challenging if a systematic sensory assessment is not performed beforehand. Another possible challenge is finding an intervention that can be performed in resource-limited settings. Another study by the present authors in this center which is pending publication has explored structured physical activity as a possible intervention and found it to improve issues in sensory processing and behavior (Original Study: Sivapalan et al.. The Effect of Structured Physical Activity on Behavior, Sensory Issues, and Skill Acquisition among Autistic Children: An Institution-based Interventional Study at a Center for Neurodevelopmental Disorders in Northern Sri Lanka; 2024).

While the current study shows that autistic children present with varied patterns of sensory issues and that sensory issues influence the severity of ASD and behavioral issues, it is limited by its inability to ascertain whether interventions targeting sensory issues can bring about changes in the severity of ASD and behavioral issues. This is an avenue future studies could explore. Furthermore, the small sample size of the study is a major limitation. This study did not have sufficient data to assess how sensory issues affect the severity of ASD and the associated behavioral problems among autistic children under three years of age. This is another area that needs exploration, as it would aid in the understanding of the impact of sensory issues in this age group and in the subsequent formulation of appropriate sensory interventions.

## Conclusions

The understanding of ASD has greatly improved over the years, and the multiple assessments and interventions have emerged to tackle various aspects of ASD. However, it appears that sensory issues have not been given due importance in the process of understanding children’s limitations, difficulties, distress, and frustrations. This study clearly shows that sensory issues among autistic individuals are varied and are associated with increased severity of ASD and increased behavioral issues. This warrants the inclusion of proper sensory assessment during the initial assessment of autistic individuals and the practice of individualized, tailor-made interventions targeting the recognized sensory issues.

## Figures and Tables

**Figure 4:**
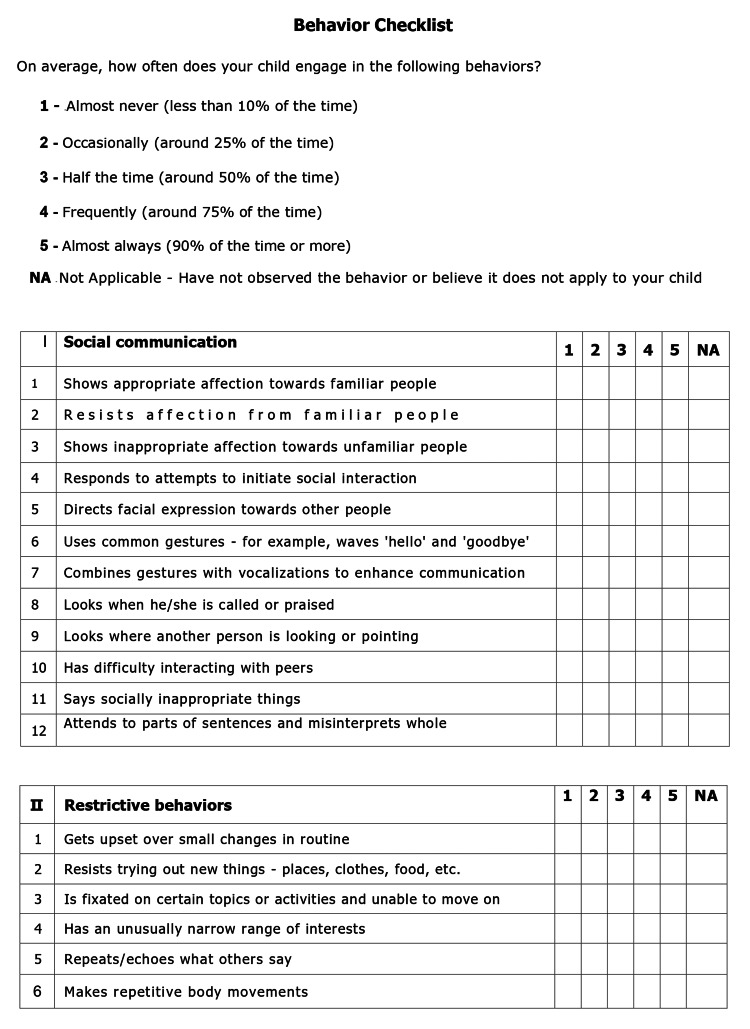
Behavior Checklist, page 1

**Figure 5:**
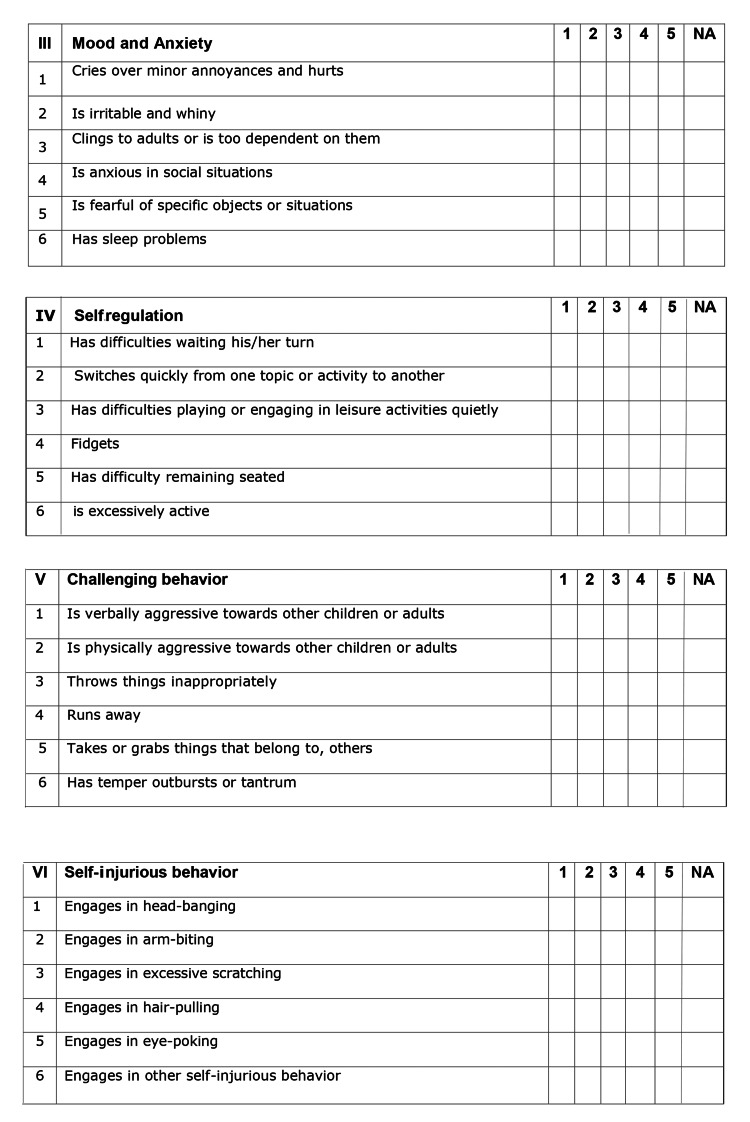
Behavior Checklist, page 2
